# Behavioral analysis in mice deficient for GAREM2 (Grb2-associated regulator of Erk/MAPK subtype2) that is a subtype of highly expressing in the brain

**DOI:** 10.1186/s13041-019-0512-x

**Published:** 2019-11-12

**Authors:** Tasuku Nishino, Kota Tamada, Akane Maeda, Takaya Abe, Hiroshi Kiyonari, Yasuhiro Funahashi, Kozo Kaibuchi, Toru Takumi, Hiroaki Konishi

**Affiliations:** 1 The Faculty of Life and Environmental Sciences, Prefectural University of Hiroshima, 5562 Nanatsuka, Shobara, Hiroshima, 727-0023 Japan; 2 RIKEN Brain Science Institute, Wako, Saitama, 351-0198 Japan; 3 Laboratory for Animal Resources and Genetic Engineering, RIKEN Center for Biosystems Dynamics Research, 2-2-3 Minatojima Minami-machi, Chuou-ku, Kobe, 650-0047 Japan; 4 Department of Cell Pharmacology, Nagoya University Graduate School of Medicine, Nagoya, Aichi 466-8550 Japan; 5 Department of Physiology and Cell Biology, Kobe University Graduate School of Medicine, Chuo, Kobe, 650-0017 Japan

**Keywords:** Adaptor protein, Tyrosine phosphorylation, Behavior tests, Neuron, Knockout mouse, Brain function

## Abstract

Grb2-associated regulator of Erk/MAPK (GAREM), is an adaptor protein related to the several cell growth factor receptor-signaling. The GAREM family has two subtypes, GAREM1 and GAREM2, both encoded in the human and mouse genome. Recent genome-wide research identified *GAREM2* as a candidate of neurodegenerative diseases. Here, we use knockout (KO) mice to show the role of GAREM2, that is highly expressed in the brain. According to the comprehensive behavioral battery, they exhibited less anxiety both in elevated plus maze and open field tests, mildly increased social approaching behavior in the reciprocal social interaction test, and longer latency to immobility in the tail suspension test as compared to wild-type (WT). Additionally, the extension of neurites in the primary cultured neurons was suppressed in ones derived from GAREM2 KO mice. Furthermore, we also identified Intersectin, as a binding partner of GAREM2 in this study. Intersectin is also a multi-domain adaptor protein that regulates endocytosis and cell signaling, which can potentially alter the subcellular localization of GAREM2. The important molecules, such as the neurotrophin receptor and Erk family, that are involved in the signaling pathway of the neural cell growth in the mouse brain, have been reported to participate in emotional behavior. As GAREM plays a role in the cellular growth factor receptor signaling pathway, GAREM2 may have a common role related to the transduction of Erk signaling in the higher brain functions.

## Introduction

Adaptor proteins mediate protein–protein or protein–phospholipid interaction through their representative functional domain such as SH2 (Src-homology 2), SH3, pleckstrin homology, and phosphotyrosine binding domains [1, 2]. These interactions are indispensable for signaling pathways of various cellular growth factors at the plasma membrane to the nucleus [3, 4]. Malfunction of some adaptor proteins by genetic alteration are closely linked to human disease; thus, numerous adaptor proteins have been identified and characterized for their physiological functions. Among these, Grb2 (growth factor receptor-bound protein 2), Gab (Grb2-associated-binding protein) and IRS1 (insulin receptor substrate 1) have been characterized precisely. They play significant role in physiological functions such as cell growth and differentiation, and are also implicated in the pathology of several human diseases [5–10]. In mammals, the Gab and IRS families include subtypes with functional differences, classified as per their expression in various organs [11–13]. The three subtypes of Gab 1–3 are widely expressed in various mammalian tissues. Using individual Gab knockout (KO) mice, abnormal Gab protein signaling has been linked to cancer, cardiovascular disease, and inflammatory disorders [14–17]. As compared to over 20 years of work on these adaptor proteins, Grb2-assocaiated regulator of Erk/MAPK (GAREM) family that we found has been analyzed in this decade [18, 19]. Therefore, precise characteristics and aspects of this family are still unknown.

Previously, we performed proteomic analysis of tyrosine phosphorylation using the epidermal growth factor (EGF)-treated A431 cell line, which highly expresses EGF receptor (EGFR) in human epidermoid carcinoma [20–22]. GAREM1 (or FAM59A) was originally identified as tyrosine phosphorylated protein in our phosphoproteomics studies, expressed ubiquitously in human tissues and a wide variety of cultured cells [18]. Then, the homologue of this molecule, GAREM2 (or FAM59B) that is highly expressed in mammalian brain tissue was also studied [19]. Even from the analysis using the cultured cells, the molecular mechanism of GAREM family involved in growth factor signaling has been relatively well-studied by biochemical and cell biology approaches.

In the growth factor-stimulated cells, the binding of GAREM to SH3 domain of Grb2 is mediated through its proline-rich motif, and to SH2 domain of Shp2 (Src homology 2-containing protein tyrosine phosphatase 2) via its tyrosine phosphorylated ITIM (immunoreceptor tyrosine-based inhibitory motifs) [23, 24]. Consequently, the expression of GAREM has an effect on regulating the activation of Erk.

In addition to these common characters of both GAREM subtypes, subcellular localization of each subtype was different, depending on the nuclear localization sequence, as seen in only GAREM1. Furthermore, the mechanism of GAREM1-specific nuclear localization cooperated with its binding protein, 14–3-3ε has been characterized [25]. However, the physiological functions of each GAREM subtype in human and mouse body are still obscured.

Unlike biochemical studies, however, smart innovation in biological and biomedical research, especially in genome-wide screening technology that uses new- generation sequencers, have revealed numerous novel genes related to various diseases [26–28]. On the meanwhile, *GAREM2* has been identified as a new positive effecter related for two neurodegenerative diseases–Alzheimer’s and Huntington’s disease [29].

To elucidate the physiological functions of GAREM2 and its relationship with human diseases, studies using its KO mice are necessary. In this study, we generated GAREM2-difficient mice and conducted comprehensive behavioral battery.

## Materials and methods

### Generation of KO mice

**Fig. 1:**
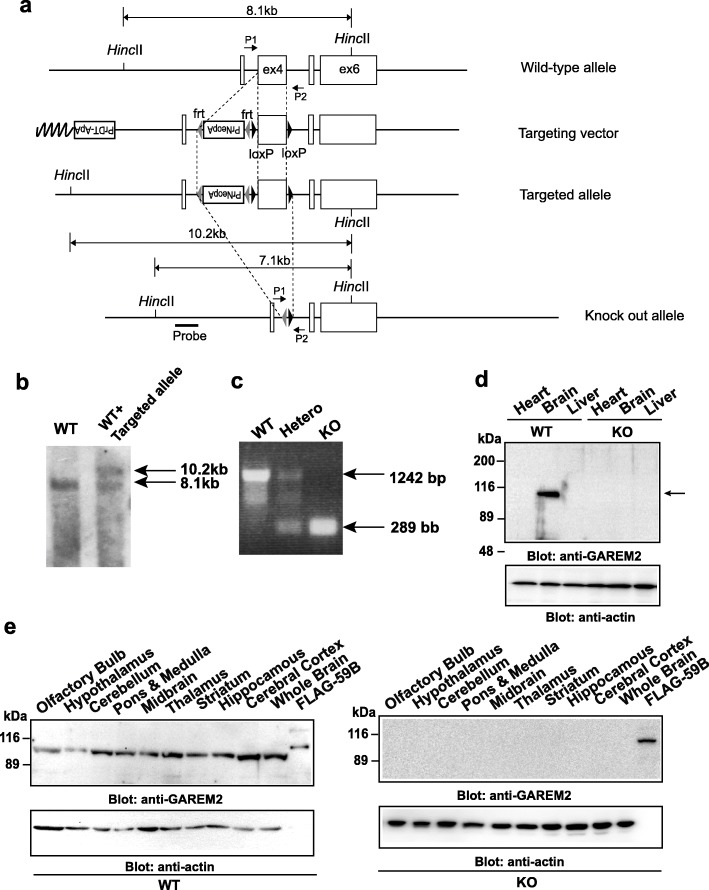
Disruption of the GAREM2 gene in mice. **a** Schematic representation of the GAREM2 targeting vector, the mouse GAREM2 gene, the targeted allele, and the deleted allele. A neomycin-resistance gene with a Pgk1 promoter and polyadenylation signal (PrNeopA), FRT sequences, and the loxP sequences are shown by open boxes, dark triangles, and filled triangles, respectively. PrDT-ApA is a diphtheria toxin A fragment gene with a MC1 promoter and rabbit β-globin gene poly A signal for negative selection [50 = 30]. Positions of probes used for Southern blotting analyses with *Hinc*II are also shown. The targeted alleles after Cre-recombination are shown at the bottom. **b** Southern blotting analysis of mouse tail genomic DNA of WT and chimeric mouse with the targeted allele. The 8.1 kbp band corresponds to the WT allele, and the 10.2 kbp band corresponds to the targeted allele involving the neomycin gene. **c** Genotyping mice by PCR after mating with CAG-Cre transgenic mouse. Mice or MEF were routinely genotyped by PCR using two primers {P1 and P2}. The P1/P2 primer set yielded the 1242 bp product from the WT and the 289 bp product from the mutant allele. **d** Characterization of mouse GAREM2 protein. Detection of endogenous mouse GAREM2 with anti-GAREM2 antibody. Protein expression of GAREM2 (upper), and β-actin (lower) in mouse tissues. Endogenous GAREM2 in the WT lysate is indicated by an arrow. Total cell lysate (20 μg) of heart, brain, and Liver from male WT and KO mice at 8 weeks of age were separated by SDS-PAGE and analyzed by immunoblotting with each antibody (right panels). **e** Expression of GAREM2 in nine brain regions. The nine brain regions were dissected and sampled from adult WT (*left*) and KO (*right*) male mice, and the total cell lysates were separated by SDS-PAGE and immunoblotted with an β-actin loading control (bottom panel). FLAG59Bfull indicates the total cell lysate of COS-7 cells transfected with FLAG-tagged GAREM2, which was immunoprecipitated with anti-FLAG antibody and used as a positive control

The GAREM2 conditional KO mice (Accession No. CDB1256K; http://www2.clst.riken.jp/arg/mutant%20mice%20list.html) were generated as described (http://www2.clst.riken.jp/arg/methods.html). The mouse GAREM2 gene comprises 6 exons located in chromosome 5 B1. The targeting vector was designed to delete exon4 containing the proline-rich region with a frt/PGK-Neo-pA/frt/loxP/exon4/loxP cassette (http://www2.clst.riken.jp/arg/cassette.html), and the targeting vector was constructed as described (http://www2.clst.riken.jp/arg/protocol.html). Southern blot analyses with a 5′ probe were performed using genome DNA derived from wild-type (WT) TT2 ES cells [30] and homologous recombinant clones. Next, 10.2 k base pairs (kbp) from a WT and 8.8 kbp from a mutant were analyzed by Southern blotting using a digoxigenin labeling and detection system (Roche). Chimeric mice were obtained from two distinct clones and mated with C57BL/6 J female mice. The heterozygous offspring were identified by genomic PCR and Southern blotting analysis with a 5′ probe as indicated in Fig. 1a. To delete the region of the target genomic GAREM2 gene (exon 4) between both loxP sequences, we crossbred the heterozygous mice with CAG-Cre mice [31] to create global knockout GAREM2 mice. Following this, we crossbred the heterozygous mice with CAG-FLP mice [32] to delete the neomycin-resistance gene between both FRT sequences from the germline. These offspring were identified by genomic PCR (5′- GACAGCTTAAGAGGAAGGGACTGG-3′; forward primer: P1, 5′- CACGGAGCCTCCGTGGTC-3′; reverse primer: P2). The expected sizes of DNA fragments were 1242 bp from the WT and 289 bp from the mutant in genomic PCR experiments (Fig. 1b).

All protocols for the animal use and experiments were reviewed and approved by the Animal Care Committees of the Prefectural University of Hiroshima, and the Institutional Animal Care and Use Committee (IACUC) of RIKEN Kobe, and Wako Branch. All efforts were made to minimize animal suffering. Mice were housed under specific pathogen-free (SPF) conditions at 24 ± 2 °C in a room with a 12-h light/dark cycle (light on at 8:00 a.m. and off at 8:00 p.m.) and had access to autoclaved food and water ad libitum.

### Behavioral test battery

#### Animals and experimental design

**Fig. 2:**
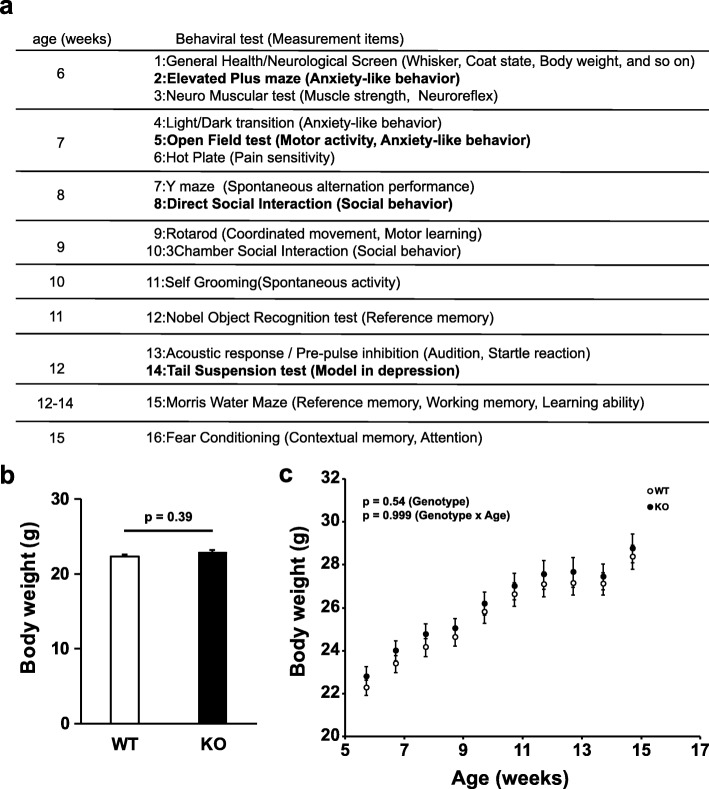
Behavioral battery list and overview of the results in GAREM2 KO mice. **a** The test name, measurement items for each test, and the date and age of the mice are shown. The results with difference in trend (*p* < 0.05 and q < 0.1) between WT and KO mice are indicated in bold. The results with no significant difference are shown in Additional file 2. **b** GAREM2 KO mice showed normal general health condition (GHNS). Graph showing the body weights of GAREM2 KO and WT mice. The body weight of the mice was measured, and the state of whiskers and coat were also noted. **c** Body weight changes in GAREM2 KO mice and WT mice during the behavioral test battery. *N* = 16 for each genotype. Error bars indicate SEM, and two-way repeated measures ANOVA was used for statistical analysis

The mice used for the behavioral test battery were generated by in vitro fertilization (IVF) after backcrossing for next 10 generations using the C57BL/6 J reference strain. All experimental mice were male GAREM2 KOs, and WT littermates were used as the controls. They were housed as two pairs of WT and GAREM2 KO mice. The behavioral test battery was performed for the screening of mouse behavioral phenotype and comprised the order that is thought that there is less stress to give mice (Fig. 2a) [33]. Unless otherwise noted, all behavioral tests performed in sound proof rooms, at about 100 lx and the mice were habituated for at least 30 min in the test rooms before all behavioral tests. After each session, the apparatuses were carefully cleaned with a solution of 70% ethanol or water to remove any odor cues.

#### General health and neurological screen (GHNS)

A general health and neurological screen were conducted, as previously described [34]. The experiment was a somatometric test that allowed the researchers, to practice mice handling, as well as allowing habituation of the mice to the researchers. We investigated the state of the whiskers, coat (presence of bald patches), righting reflex, ear twitch response and body weight of the mice.

#### Elevated plus maze test

The experiment was performed to assess anxiety-like behavior of mice and was conducted as previously described [35, 36]. The elevated plus maze consisted of two open arms (25 × 5 cm) and two enclosed arms of the same size, with 15 cm high transparent walls. The arms and central square were made of white plastic plates and were elevated to a height of 50 cm above the floor. To minimize the likelihood of animals falling from the apparatus, 5 mm high Plexiglas ledges surrounded the open arms. Arms of the same type were arranged at opposite sides to each other. At first, each mouse was placed in the central square of the maze (5 × 5 cm) facing the left side enclosed arms. Time spent in each of the arms was recorded for 10 min. Data acquisition and analysis were performed automatically using Image EP software (Time EP2 for Elevated Plus Maze, O′ Hara & Co., Tokyo, Japan).

#### Open field test

Locomotor activity of mice was measured using an open field (OF) test. Each subject was allowed to move freely in the open field apparatus (50 × 50 × 30 cm; O’Hara & Co.) equipped with photocells (beam spacing 2.5 cm, beam diameter 4 mm, beam frequency 50 Hz) for detecting vertical activity of subject mouse. Total distance traveled, vertical activity (rearing measured by counting the number of photobeam interruptions), time spent in the center area of the open field were recorded for 120 min by Time OFCR4 (O’hara & Co.). The center area was defined as an inner section whose perimeter was 10 cm away from each of the walls. Anxiety index was calculated as the time spent in the center area: total distance ratio in first 15 min [36].

#### Direct social interaction test (one-chamber social interaction test)

The reciprocal social interaction test was performed with a modified procedure from a previous study [37, 38]. Firstly, habituation to the open field was performed for each mouse, 10 min prior to the test session. Two mice of identical genotypes housed in different cages were placed in a chamber (50 × 50 × 50 cm; O’Hara & Co.) with dim light (10 lx) and were allowed to move freely for 10 min. These two mice were not only identical of genotype but also of same age and sex and, had similar body weight. In this test, a pair of mice was used as a sample. Images were captured at the frame rate of two per second by a CCD camera located above the field. If the two mice contacted each other or stayed near, it was defined as “Direct interaction time” and “Approached time”, respectively. To avoid observer bias, the analysis of each time was performed automatically using homemade macro in the ImageJ software.

#### Tail suspension test

The tail suspension test was performed to evaluate mouse depressive-like behavior [39]. At first, the subject mouse tail was fixed on the metal plate with adhesive tape, which was placed approximately 1 cm from the tip of the tail and suspended 30 cm above the floor in a visually isolated chamber. Immobile behavior was recorded for 10 min. Data acquisition and analysis were performed automatically using Image TS software (O’Hara & Co.). Latency to the first bout of immobility was defined as more than 5 s segment of time spent immobile [40].

#### Statistical analysis

Behavioral data were obtained automatically by customized software (O’Hara & Co.). Statistical analysis was conducted using StatView Ver 5.0 (SAS Institute, Cary, NC, USA). Data were analyzed by a two-way analysis of variance (ANOVA), two-way repeated-measures ANOVA, or unpaired t-test. Unless otherwise noted, *p* values donate the genotype effect. The criterion for significance was set at *p* < 0.05.

### Neuronal primary culture of mouse hippocampus

Hippocampal neurons were obtained from the embryonic brains at embryonic age 17.5 days (E 17.5) of GAREM2 heterozygous female mice that were bred with GAREM2 heterozygous male mice. The hippocampi of the embryonic brains were dissected briefly in calcium and magnesium free Hank’s Buffered Salt Solution (CMF-HBSS) on ice under an optical microscope (SZX7, Olympus, Tokyo, Japan). The hippocampi were dissociated with Neuron Dissociation Solutions (code: 291–78,001, Wako, Tokyo, Japan) according to the manufacturer’s protocol (www.sumibe.co.jp/ww-attaches/110.pdf). The dissociated neurons were seeded in Neural Plating Medium (NPM), Minimum Essential Media (MEM; Gibco) containing 5% fetal bovine serum (FBS), 2 mM L-glutamine, 0.6% D-glucose, 100 U/mL penicillin and 100 μg/mL streptomycin. Between 4 and 6 h after seeding, the neurons were cultured in a humidified incubator at 37 °C and 5% CO_2_. The medium was changed to Neuronal Maintenance Medium (NMM), neurobasal containing 1x MACS NeuroBrew-21 (50× stock solution, Miltenyi Biotec), 0.5 mM L-glutamine, 100 U/mL penicillin and 100 μg/mL streptomycin. The neurons were cultured in an incubator.

### Analysis of neurite outgrowth

To measure the length of neurites, and to maintain the viability of the hippocampal primary cultured neurons, the neurons were seeded on to coverslips in 12-well plates that were pre-coated with poly-L-lysine (PLL) (diluted to 0.1 mg/mL with 0.1 M borate buffer) (Sigma) at a density of 5.0 × 10^4^ cells/well. The dissociated cells were counted by using the TC20 Automated Cell Counter (Cat.NO.#145–0101, BioRad). On in vitro day 3 (DIV3), cells were fixed with 4% paraformaldehyde and 2% sucrose in 0.1 M phosphate-buffered saline (PBS) and then processed for immunocytochemistry. The immunostained neurons were observed and scanned using the VS120-S5-J slide scanner (Olympus) and converted into each image to analyze neurites using OlyVIA 2.9. (Olympus) software and ImageJ. The length of neurite was measured with NeuronJ [41]. Statistical analysis was conducted using StatView Ver. 5.0.

### Immunocytochemistry and confocal microscopy analyses

Neurons were fixed with 4% paraformaldehyde/0.1 M PBS for 15–20 min at room temperature. The cells were washed twice with 0.1 M PBS and three times 0.3% Triton X-100 diluted with 0.1 M PBS (PBST). Cells were blocked with 5% normal goat serum diluted with PBST for 1 h at room temperature. The neurons were incubated with primary antibodies diluted with the blocking buffer overnight at 4 °C. The primary antibodies used for this study were anti-Map 2 chicken polyclonal antibodies (1:5000) (Cat. No. ab5392, Abcam, UK) and anti-Tau rabbit polyclonal antibodies (1:500) (Cat. No. ab64193, Abcam). The neurons were washed with PBST three times and then incubated with secondary antibodies diluted with the blocking buffer for 2 h at room temperature. The secondary antibodies used for this study were the anti-chicken goat IgY conjugated with Alexa Fluor 488 (1:1000) (Cat. No. ab150169, Abcam) and anti-rabbit goat IgG conjugated with Alexa Fluor 568 (1:500) (Cat. No. A-11019, Invitrogen, USA). The cells were washed with PBST three times, 0.1 M PBS three times, and 0.1 M phosphate buffer once. Following this, the neurons seeded on the coverslips were mounted on glass slide using VECTASHIELD Mounting Medium with DAPI (Cat. No. NC9524612, Vector Laboratories, USA).

To express GFP-fused intersectin2 (ITSN2) [42] in the cultured cells, the full-length human GAREM2 cDNA was inserted into a pEGFP-C3 vector [19] was described previously. Transfected and non-transfected cells were fixed with 5% formaldehyde in phosphate-buffered saline (PBS) for 10 min, washed with PBS, permeabilized with 0.1% Triton X-100 in PBS for 10 min, and then washed with PBS once more. Following a blocking step with 3% bovine serum albumin in PBS for 30 min, the primary antibodies, as illustrated in the Fig. 6, were applied for 1 h. After washing with PBS, the cells were incubated with the appropriate secondary antibodies conjugated with Alexa fluorescent dyes (Molecular Probes) for 45 min. If necessary, the nuclei were simultaneously stained with 2 μM Hoechst 33342 (Molecular Probes). Finally, the cells were rinsed three times with PBS and mounted onto microscope slides with ProLong Antifade reagents (Molecular Probes). Images were captured using an Olympus BX51 fluorescence microscope or an FV10i-LIV laser scanning confocal microscope with a 10, and 60 × 1.0 numerical aperture Plan Apo objective. The figures were prepared using Adobe Photoshop.

### Immunoprecipitation and immunoblot analysis

The following procedures were carried out at 0–4 °C. The mouse brains (dissected to eight regions (Fig. 1e) in 1× Hank’s Buffered Salt Solution) and the cultured cells were lysed in a lysis buffer containing 20 mM Tris (hydroxymethyl) aminomethane (Tris)-HCl (pH 7.5), 1 mM EDTA, 10 mM dithiothreitol (DTT), 1% Triton X-100, 150 mM NaCl, 10 mM NaF, 1 mM Na_3_VO_4_, and a complete protease inhibitor cocktail (Roche) to produce a total-cell lysate (TCL). For the immunoprecipitation experiments, the TCL was centrifuged, and the supernatant was incubated for 2 h with either the mouse GAREM2 antibody or an anti-FLAG affinity gel (Sigma). Protein G-Sepharose (GE Healthcare Life Sciences) was added, and the resulting mixture was rotated at 4 °C for 1 h. The beads were subsequently washed three times with the lysis buffer. The processed samples were boiled in sodium dodecyl sulfate (SDS) sample buffer, separated by SDS-polyacrylamide gel electrophoresis (PAGE), and transferred onto an Immobilon-P membrane (Millipore). Immunoblot analysis was carried out using primary antibodies, as described in the figure legends. Immunoreactive bands were visualized using horseradish peroxidase-conjugated anti-rabbit or anti-mouse IgG and ECL reagent (GE Healthcare Life Sciences).

### Reagent, cell culture, antibodies and transfection

COS-7 and SH-SY5Y cells were maintained in Dulbecco’s modified Eagle’s medium (DMEM) supplemented with 10% FBS, 100 μg/mL streptomycin, and 100 U/mL penicillin. Plasmid transfection into COS-7 cells was carried out by electroporation using a Gene Pulser (Bio-Rad). Preparation of the polyclonal anti-GAREM2 has been described previously [19]. The anti-FLAG M2, anti-ITSN1 and 2 (Sigma) were obtained commercially.

### Cloning of the SH-SY5Y stable cell line

The cDNA for expression of the N-terminal FLAG-tagged GAREM2 was inserted into pQCIXP (TAKARA-Clontech), and the resulting plasmid was used for generating retrovirus expression vector. The empty vector was used as a control. The packaging cells (AmphoPackTM-293 cell line; TAKARA-Clontech), were transfected with the appropriate retroviral expression construct by electroporation. Culture supernatants were collected after 48 h post-transfection and centrifuged. SH-SY5Y cells were infected with the viral supernatants in the presence of 8 μg/ml Polybrene (Sigma) for 12 h, after which the medium was changed. Following infection, cells were selected with 2 μg/ml puromycin.

### Statistical analysis

The data obtained in this study are expressed as mean ± standard error (S.E.). Statistically significances of the differences were determined by comparing between groups using one-way analysis of variance (ANOVA) and the Tukey-Kramer test. A *P*-value < 0.05 was regarded as significant. To control type I error in behavioral battery, we calculated q value which is an extension of a quantity called the false discovery rate as a multiple hypothesis correction [43]. Each q value was shown in figures when *p* value was less than 0.1. All statistical results were shown in Additional file 1: Table S1.

## Results

### Generation of conditional GAREM2 KO mice

To investigate the role of *GAREM2* in mice, we generated a conditional null allele of GAREM2 gene. Targeting of GAREM2 *gene* was performed by introducing a frt/PGK-Neo-pA/frt/loxP/exon4/loxP cassette into intron 2 *and loxP* sites into intron 4 regions through homologous recombination in mouse TT2 ES cells (Fig. 1a). Germline transmission was confirmed by PCR and Southern blotting analysis (Fig. 1b). We generated the global KO by mating *GAREM2*-floxed mice with CAG-Cre transgenic mice, which express Cre recombinase ubiquitously, allowing for the generation of *GAREM2* ^+/−^ mice. After deleting the neomycin-resistance gene by crossbreeding with CAG-FLP mice, heterozygous mice were then intercrossed to produce homozygotes. The mutation deleted a region including the neomycin-resistance gene and exon4 was confirmed by genomic PCR (Fig. 1c). GAREM2 KO mice were viable and fertile, and showed no obvious differences compared with WT mice in the 12 weeks following birth. We have previously reported that GAREM2 is highly expressed in the brain. Using mouse GAREM2-specific antibody, we detected a GAREM2 protein band in the lysate of whole brain of mouse but not in that of KO mouse (Fig. 1d). To elucidate the expression of GAREM2 in detailed regions of the brain, we performed western blotting analysis. The brain of adult WT male mice were dissected into nine regions and the total lysates were used for immunoblotting with an anti-GAREM2 antibody. The results suggested that the GAREM2 protein may be expressed in all nine regions of the brain (Fig. 1e).

GAREM2 KO mice were born without complications and exhibited normal physiological development. However, we observed that the KO mice were sensitive to sounds and objects, e.g., researchers’ hands, in comparison with other mice. Therefore, we performed mice behavioral analysis (behavioral test battery) with the GAREM2 KO mice to characterize the phenotype of its behavior.

### GAREM2 KO mice showed normal general health

Figure 2a shows the list and the summary of the results of mouse behavioral test battery performed in this study.

Sixteen male mice representing each genotype were used. The body weight evaluations suggested that GAREM2 KO and WT mice had almost the same body weight (Fig. 2b). No physical abnormalities were seen in any mice. Therefore, the effect of knocking out *GAREM2* did not bring about any physical abnormalities in mice. Moreover, the body weights of GAREM2 KO and WT mice during the behavioral test battery were similar (Fig. 2c).

### GAREM2 KO mice exhibited reduced anxiety-like behavior in the elevated plus maze (EPM) test, and tended to be higher social interaction

**Fig. 3:**
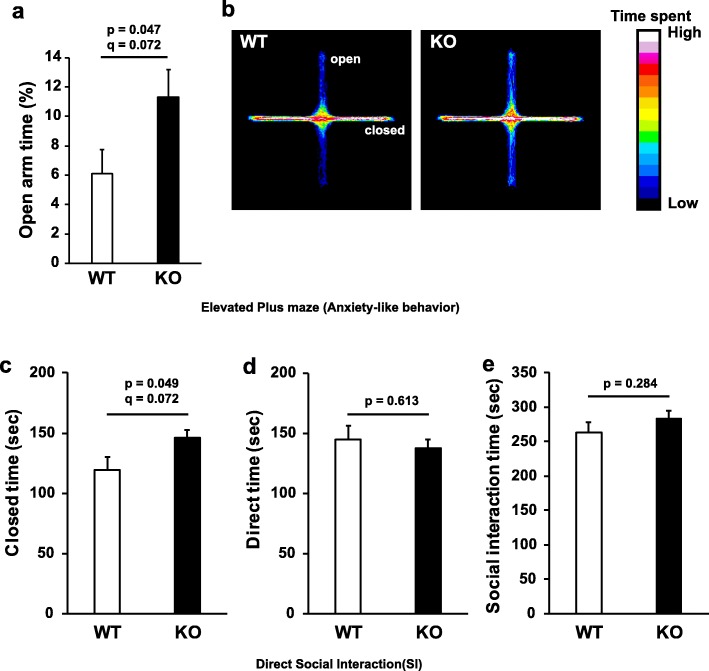
GAREM2 KO mice exhibited reduced anxiety-like behavior in EPM test (**a** and **b**). **a** Graph showing that the time (%) spent in open arms in GAREM2 KO mice was higher than WT mice. N = 16 for each genotype. Error bars indicate SEM. **b** Heat maps show the time spent in open and closed arm in WT (left panel) and GAREM2 KO mice (right panel). GAREM2 KO mice exhibited a tendency to be close to each other in the direct social interaction test. **c**-**e** Graphs showing the closed time, direct interaction time, and social interaction time (sec), respectively in WT and GAREM2 KO mice. In the direct social interaction test, the combined total of closed time and direct interaction time was denoted as “social interaction time”. *N* = 8 pair for each genotype and error bars indicate SEM

In an EPM test, the longer time spent in the open arms indicated the low anxiety levels in mice. The time (%) spent in the open arms by GAREM2 KO mice was trend to be increased compared to WT mice (Fig. 3a and b). Our results of the EPM test suggested that, GAREM2 KO mice demonstrated lower anxiety compared with WT mice.

In the direct social interaction test, the combined total of closed time and direct interaction time and was denoted as “social interaction time”. It can be seen that, GAREM2 KO mice tended to have longer periods of closed time than WT mice (Fig. 3c). However, the two groups did not differ in the direct time and social interaction time (Fig. 3d and e). Therefore, GAREM2 KO mice had mildly high social approaching behavior.

### GAREM2 KO mice exhibited increased exploratory activity and reduced novelty-induced anxiety

**Fig. 4:**
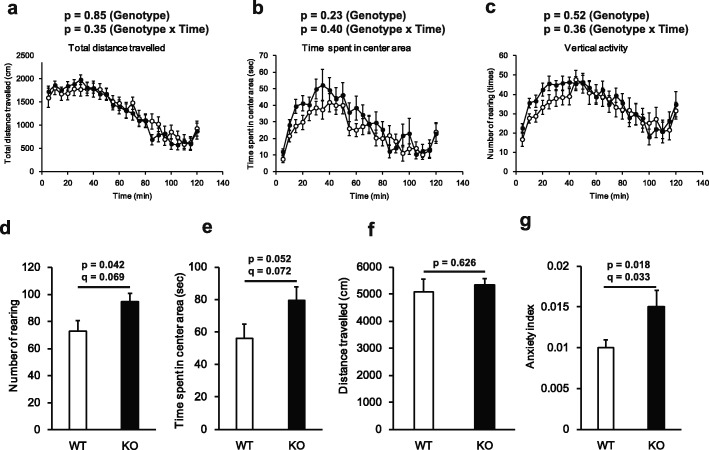
GAREM2 KO mice showed hyper exploratory activity and low novelty-induced anxiety in the open field test. Total distance travelled, time spent in the center area, and the number of rearing incidents for the total duration (120 min) were analyzed in the open field test. **a** The change of the total distance travelled (cm) for each genotype. **b** The change of the time spent in the center area (sec) for each genotype. **c** The change of the number of rearing behaviors (times) for each genotype. **d**-**g** Graphs showing the number of rearing behaviors, time spent in the center area, distance travelled, and anxiety index for GAREM2 KO and WT mice in the analysis of the first 15 min. Anxiety index (=center time / distance travelled). N = 16 for each genotype, error bars indicate SEM

The measured parameters of total travelled distance, time spent in the center area and rearing number for total duration time (120 min) were not shown to differ greatly between GAREM2 KO and WT mice in the open field test (Fig. 4a-c). However, the analysis of the first 15 min, often regarded as the most important period in a new environment, GAREM2 KO mice had a trend to spend longer time in the center area, and the number of the KO mice rearing was more than that of WT mice (Fig. 4d and e). In the open field test, the time spent in the center area and rearing number are used as indexes for novelty-induced anxiety and exploratory locomotor activity, respectively. In addition, we utilized the ratio of center/total locomotor activity to assess anxiety. GAREM2 KO mice presented a higher anxiety index compared to the WT mice (Fig. 4g). In the open field test, the higher anxiety index indicates the lower the anxiety in the mouse. Consequently, GAREM2 KO mice were more active and exhibited lower anxiety than the WT mice.

### GAREM2 KO mice exhibited longer latency to immobile behavior than WT mice

**Fig. 5:**
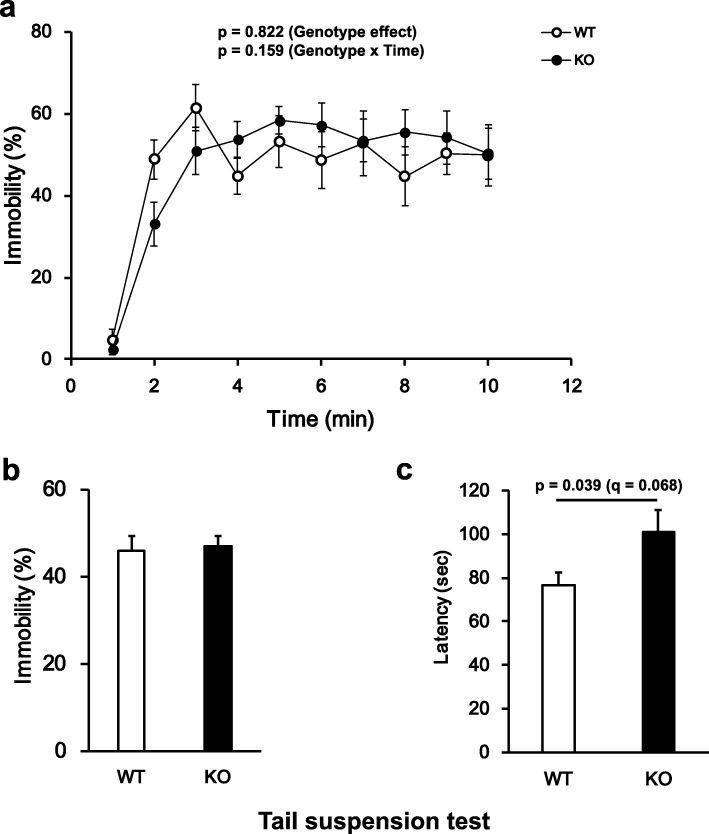
GAREM2 KO mice exhibited longer latency to immobile behavior than WT mice in the tail suspension test. **a** The average of the immobility rate (%) per min for WT and GAREM2 KO mice. A two-way repeated measures ANOVA was used. **b** Graph showing the immobility (%) for each genotype. **c** Graph showing the analyzed latency to immobility (sec) in the first 2 min analysis. *N* = 15 for WT, 15 for KO, error bars indicate SEM

GAREM2 KO mice were subjected to the tail suspension test, a mouse behavior test for validating the susceptibility for depressive stress. We verified depression like behavior by two parameters including total time of immobility and latency to first bout of immobile (5 s <). No significant differences in immobility (%) were detected between the two genotypes (Fig. 5a and b). However, the latency to the first incidence of immobility in GAREM2 KO mice showed a tendency to be longer than in WT mice (Fig. 5c).

### GAREM2 regulated neurite outgrowth, and effects of ITSN on the subcellular localization of GAREM2

**Fig. 6:**
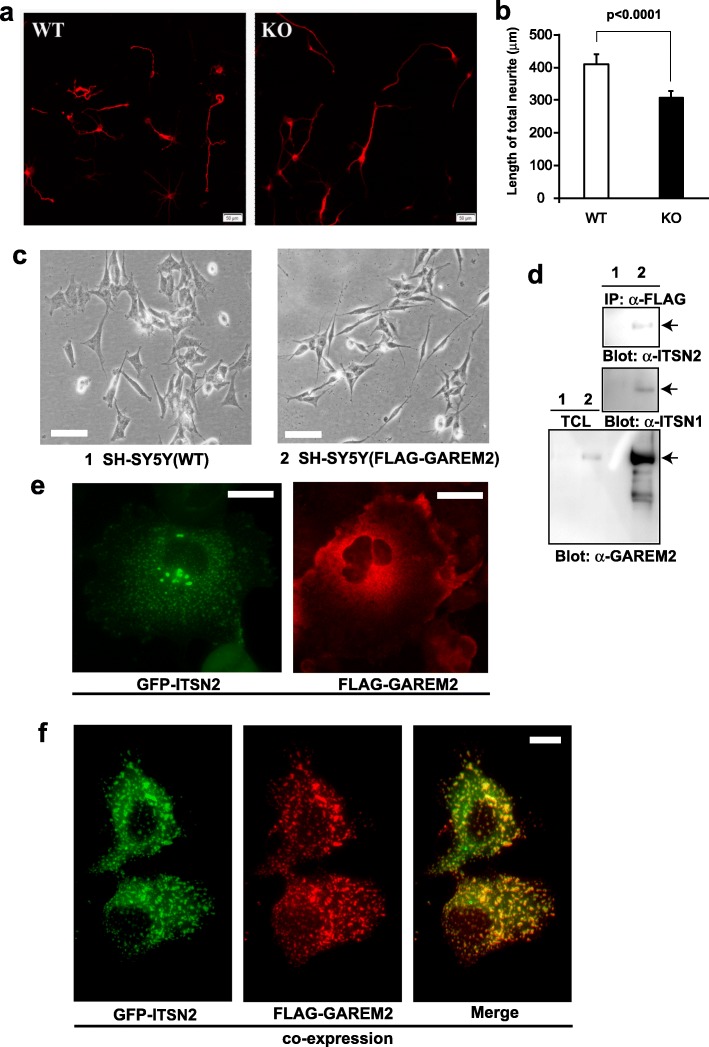
GAREM2 may work as a regulator of neurite outgrowth in neurons. **a** The primary cultured hippocampal neurons (DIV 3) were immunostained with an anti-Tau antibody. The representative images for WT (left panel) and GAREM2 KO neurons (right panel). **b** Graph showing the length of total neurites for each genotype. The total neurite consists of all axons and all dendrites. Error bars indicate SEM. *Scale bar*, 50 μm. **c** Expression of FLAG-GAREM2 induced morphological alteration of SH-SY5Y cells. Images show representative 40–50 cells of the SH-SY5Y cell line. Images were taken at 48 h of the last passage of the cells in the culture dish containing normal growth medium. Images of phase contrast of control SH-SY5Y (1: *left*) and the A431 clone stably expressing FLAG-GAREM2 (2: *right*) are shown. *Scale bar*, 50 μm. **d** Association between GAREM2 and both ITSN1 and ITSN2 in SH-SY5Y cells. Immunoprecipitation (*IP*) experiments were carried out with total cell lysates (TCL) of the SH-SY5Y cells lysates using anti-FLAG antibody. Immunoblot (*IB*) analysis was carried out with anti-ITSN2 (*top*), anti-ITSN1 (*middle*), and anti-FLAG (*bottom*) antibodies. **e** Subcellular localization of FLAG–GAREM2 (red) and GFP–ITSN2 (green). COS-7 cells expressing FLAG–GAREM2 (*right*), GFP–ITSN2 (*left*). **f** COS-7 cells co-expressing both FLAG–GAREM2 (*middle*) and GFP–ITSN2 (*left*). FLAG-GAREM2 proteins (red) were processed for immunofluorescence staining using anti-FLAG antibody. The merged image is shown on the *right*. *Scale bar*, 10 μm

According to behavioral tests, loss of GAREM2 causes some emotional changes in mouse. We also performed morphological analyses in cellular level to elucidate the mechanism of these behavioral phenotypes. Although the GAREM2 protein is distributed in widespread of the mouse whole brain, apparent abnormalities of brain structure were not detected (data not shown). In the previous study, the result of knockdown experiments using small interfering RNA (siRNA) in a neuroblastoma cell line (SH-SY5Y) suggested that GAREM2 is a regulator of IGF-1-induced neurite outgrowth [19]. Therefore, we analyzed neurite outgrowth with GAREM2 KO mouse primary cultured hippocampal neurons. The total length of neurites (including axons and dendrites) in the primary cultured neurons of GAREM2 KO mice was shorter than WT neurons (Fig. 6a and b).

Grb2 and Shp2 have been identified to bind to GAREM2 in our hands. Additionally, it has been reported that GAREM2 was a candidate of the binding partner of ITSN family that is a multi-domain scaffold protein by yeast two hybrid system [44]. In the present study, we confirmed the interactions between ITSN and GAREM2 protein in mammalian cultured cells. To identify the protein binding to GAREM2 in neuronal cells, we have established the SH-SY5Y cell line stably expressing FLAG-GAREM2. Interestingly, the FLAG-GAREM2 expressing cells induced the neurite outgrowth in the normal growth medium (Fig. 6c). In these cell lines, association of GAREM2 and both ITSN subtypes was confirmed by immunoprecipitation (Fig. 6d).

According to the analysis of KO mice, ITSN1 has an essential and novel role in axonal growth at the cortical midline as well as in spatial learning [45]. ITSN is a dynamin-binding protein implicated in numerous functions of the nervous system, including synapse formation and endocytosis. Therefore, both ITSN1 and 2 are localized is in the cytosol forming an endosomal vesicle. The FLAG-GAREM2 in COS-7 cells transfected with only its expression vector was widely distributed in the cytosol (Fig. 6e). Additionally, we observed an interesting phenomenon wherein ITSN2 was colocalized with GAREM2 at the vesicle formed in COS-7 cells expressing both FLAG-GAREM2 and GFP- ITSN2 (Fig. 6f).

## Discussion

As the characteristic of each subtype of GAREM is similar, no difference in the phenotype of GAREM2 deficient mice might be expected by complementation of loss of GAREM2 by GAREM1 that is also expressed in the brain. However, comprehensive behavioral battery analysis indicated GAREM2 KO mice exhibited low anxiety-like behavior in the EPM test (Fig. 3a and b), tended to higher social approaching behavior (Fig. 3c-e), and increased exploratory activity / reduced novelty-induced anxiety in the open field test (Fig. 4). Moreover, the results of the tail suspension test also suggested that GAREM2 KO mice exhibit increased activity (Fig. 5). Immobility during the tail suspension test is used as the indicator of despair or “depression-like” behavior, whereas persistent struggling can indicate hyperactivity or manic-like responses in mice [46]. Although we could not detect the statistical difference for multiple hypothesis testing in most of these behavioral results, except for anxiety index in the open field test (Fig. 4g), GAREM2 KO mice consistently tended to be less anxiety in different tests. These phenotypical differences could be associated with the one of the roles of GAREM2. The similar phenotypes of emotional behavioral alteration have been observed in the KO mice of p75NTR [47], Braf [48], Erk 1[49] and Erk2 [50].

The results of the morphological analysis of hippocampal primary cultured neurons in GAREM2 KO mice showed that GAREM2 regulates neurite outgrowth in neurons. Recent report described that GAREM2 has been identified as a candidate molecule of neurodegenerative diseases [29]. During our observation period of the behavioral tests, no significant defects were seen in the brain structure until 20 weeks after birth (data not shown). Further studies using older mice are needed to elucidate the relationship between the function of GAREM and Alzheimer’s and Huntington’s diseases. Age- and region-dependent expression profiling of each GAREM subtype is important to understand their functions in the brain. Our biochemical studies using a specific antibody revealed that GAREM2 expresses in all regions of mouse brain. However, the immunohistochemical staining sections of the mouse brain could not be performed owing to the cross-reaction and high-background of the specific antibody against each GAREM subtype. Currently, we are attempting generate a monoclonal antibody with high specificity for each GAREM subtype for immunohistochemical experiments. We have also established GAREM1-difficient and GAREM1- and GAREM2-double KO mice, and are currently studying their phenotypes. Generation of mice deficient in both GAREM1 and GAREM2 might elucidate a possible redundancy of these two scaffolding proteins and reveal their function in mice.

Although some significant differences between WT and KO mice were seen in the behavior tests, molecular mechanism of this phenomenon is still unclear. In order to address this issue, it is critical to identify a specific binding-partner of GAREM2 in the brain. The yeast two-hybrid results from a previous study suggested that ITSN1 and 2 were binding partners of GAREM2 [44]. In this study, we demonstrated that both ITSN subtypes bind to GAREM2 in the cultured and brain cells. Furthermore, the subcellular localization of GAREM2 was dramatically influence with the ITSN expression. Therefore, this result suggests that GAREM2 and ITSN family cooperate in the function of mouse brain.

The ITSN family is one of the scaffold proteins related to the Reelin pathway to regulate neuronal migration and synaptic plasticity in hippocampus [51]. The ITSN1 KO mice and the double KO (ITSN1 and 2) mice lack corpora callosa and exhibit a fiber tract deficiency in the regions that cross the medial longitudinal fissure. The results of the behavioral test (Morris water maze) suggested that ITSN1 is significant for learning and memory [45]. Therefore, in future studies, we intend to perform morphological analysis using brain sections of GAREM2 KO mice. Furthermore, we are interested in in vivo studies to explore the relationship between GAREM2 and its binding partners, included in the ITSN family.

In addition to ITSN family, 14–3-3 family was also identified as a GAREM-binding protein [25]. As both GAREM subtypes contain the consensus phosphorylation site for binding to 14–3-3, it could have potential effects on GAREM function. In general, the 14–3-3 domain interacts with many binding partners in a phosphorylation dependent manner and regulates the function or subcellular localization of its target proteins [52]. GAREM2 have two conserved phosphorylation dependent 14–3-3-binding sites. The sequences neighboring the serine 502 (S502) residue and threonine 588 (T588) residue of GAREM2 are RLSSSP and RALTEP, respectively. These are good matches for consensus binding sites of the 14–3-3 family member, RXXphosphoS/TXP (where X is any amino acid residue) [53]. The 14–3-3 proteins were originally discovered as a family of proteins that are highly expressed in the brain, that bind and regulate several key molecules related to neurodegenerative disease such as Alzheimer’s and Parkinson’s [54]. Therefore, it is possible to speculate that 14–3-3 proteins play important roles in pathogenesis through regulating the subcellular localization of GAREM protein.

GAREM2 may have a role in higher brain functions regulating the Erk signaling pathway with its binding proteins. This study may help to elucidate the link between GAREM2 and emotional behavior, and reveal GAREM2 as a therapeutic target for neurogenerative disease.

## Supplementary information

**Additional file 1.** All results of statistical analysis in behavioral data.

**Additional file 2.** The results of behavioral battery with no significant difference.

## Data Availability

The datasets supporting the conclusion of this study are included in this article.

## Abbreviations

EGF: Epidermal growth factor
Erk: Extracellular signal-regulated kinase
GAREM: Grb2-associated regulator of Erk/MAPK
Grb2: *Growth factor* receptor-bound protein 2
MAPK: Mitogen-activated protein kinase
