# Supplementary material for: Behavioral analysis in mice deficient for GAREM2 (Grb2-associated regulator of Erk/MAPK subtype2) that is a subtype of highly expressing in the brain
Source: Mol Brain. 2019 Nov 12;12:94. doi: 10.1186/s13041-019-0512-x (PMC6852768; doi:10.1186/s13041-019-0512-x)
Supplement: Supplementary file 2 — Additional file 2. The results of behavioral battery with no significant difference. [file 13041_2019_512_MOESM2_ESM.doc]

**Supplemental data**

**Materials and methods**

*Other behavioral tests*

Following all behavior tests were conducted based on previously published reports [1, 2]

[Neuromuscular test]

Neuromuscular strength was examined by grip strength and wire hang tests. We used the grip strength meter (O’Hara & Co.) to assess the forelimb grip strength of mice. Mice were lifted and held by their tail, allowing them grasp a wire grid with their forepaws. Mice were then gently pulled backwards by the tail with their posture parallel to the surface of the table until they released the grid. The peak forelimb grip force applied by the mice was recorded in newtons [3]. Each mouse was tested three times and the greatest value measured was used for statistical analysis. In the wire hang test, mice were placed on a wire mesh that was then inverted and moved gently, so that the subject gripped the wire. Latency to fall onto the bedding within 60 sec was recorded by counting manually. Moreover, the whisker and ear twitch, righting reflex, reaching of forepaws and reaction to key jangling were evaluated. A number of physical features of mice, including malocclusion and state of eyes, were also recorded.

[Light/dark transition test]

The experiment was performed to assess anxiety-like behavior. The apparatus used for the light/dark transition test was divided into two chambers by a partition containing a door (3 × 5 cm) (O’Hara & Co.) [4]. One chamber was brightly illuminated (390 lux), whereas the other section was dark (2 lux). At first, mice were placed into the dark side of the apparatus, and allowed to move freely between the two chambers for 10 min, while the door was kept open. The total number of transitions, time spent in each chamber, initial latency to the light box, and distance traveled were recorded automatically using Image LD software (O’Hara & Co.). Online material describing this method is available [5].

[Hot plate test]

The hot plate test was used to evaluate nociception. Mice were placed on a hot plate at 55 ± 0.3 ˚C (Ugo Bsaile 7280 Hot Plate, Artisan Technology Group, USA) and latency to the first hind paw response was recorded by counting manually. The hind paw response was either a foot shake or a paw lick. Each mouse was tested once.

[Y-maze test]

Exploratory activity was working memory were evaluated by using a Y-maze apparatus (arm length: 40 cm, arm bottom width: 3 cm, arm upper width: 11.8 cm, height of wall: 12 cm). Each subject was placed in the center of the Y-maze field (50 lux). The number of entries and alternations were recorded using a modified version of the Image EP program. Data were collected for 10 min.

[Rotarod test]

Motor coordination and balance were tested using a rotarod test. The test was performed by placing a mouse on a rotating drum (3 cm diameter; ROTA-ROD TREADMILL FOR MICE MK-610A, Muromachi Kikai Co., Tokyo, Japan) and measuring the latency (sec) for each mouse to fall from the rod. Six trials (3 trials/day) were performed and the speed of the rotarod was gently increased from 4 to 40 rpm over a 5 min period.

[Three-chambered social interaction]

Social testing apparatus consisted of a rectangular, three-chambered box and a lid with an infrared video camera [6] (O’Hara & Co.). Each chamber was 20 × 40 × 22 cm, and the dividing walls were made from clear Plexiglas, with small square openings (5 × 3 cm each) allowing access into each chamber. An unfamiliar C57BL/6J male mouse (stranger mouse) that had no prior contact with subject mice was placed in one of the side chambers. The location of the stranger in the left versus right side chamber was systematically alternated between trials. The stranger mouse was enclosed in a small, round wire cage, which allowed olfactory, visual, auditory, and tactile contacts but did not allow sexual and deep contacts (e.g., not mount). The subject mouse was first placed in the middle chamber and allowed to explore the entire social test box for a 10 min session. The amount of time spent in the quadrant was measured by a camera attached to the top of the box.

[Self-grooming test]

Spontaneous activity was measured using a self-grooming test. Firstly, the test mouse was placed in a clear box (18 × 10.5 × 11 cm) and habituation was performed for 5 min (brightness: 30 lux). The test session then started, and ran for 5 min. The total grooming time of each mouse was measured manually.

[Novel object recognition test]

The novel object recognition test was performed to evaluate access to novelty and reference memory and using the procedure of a previous study [6]. Because this test requires spontaneous exploratory activity in mice, the test was conducted in the dark phase (from 9:00 pm) and with dim light (15 lux) in the chamber. First, habituation to the test chamber (50 × 50 × 50 cm) was performed for 10 min for each mouse once a day for three days. At 24 hr after the last habituation, place the two identical objects were placed in the chamber. The pair of objects (either a dice or cylinder) was randomized between each mouse and each group tested. At first, we placed the mouse in the open field chamber; its head positioned opposite the objects, and then a stopwatch was started to measure the time necessary to reach the 20 sec criterion of total exploration. The experiment was stopped when there has been a 20 sec recorded exploration of both objects or when the 10 min period was over. After 24 hr, the two familiar objects were replaced, one was replaced with the same object and the other was replaced with a novel object and they were placed at the same position. The location of the novel object (left or right) had to be randomized between each mouse and each group tested.

[Startle response/prepulse inhibition test]

Startle responses and prepulse inhibition (PPI) of the startle responses were performed using the procedure of a previous study [7] and were measured using an automatic startle reflex measurement system (O’Hara & Co.). A test session began by placing a mouse in a Plexiglas cylinder, and left undisturbed for 10 min, as a form of habituation. The startle stimulus was a broadband white noise that lasted 40 msec for all trial types, and the startle response was recorded during this time period (measuring the response every 1 msec) without the prepulse stimulus. The PPI response was recorded for 140 msec starting with the onset of the prepulse stimulus. The background noise level in each chamber was 40 dB. The peak startle amplitude recorded during the 140 msec sampling window was used as the dependent variable. A test session consisted of nine trial types (i.e. one type for non-stimuli, two types for startle stimulus only trials, and six types for PPI trials). The intensity of the startle stimulus was 110 or 120 dB. The prepulse was presented 100 msec before the startle stimulus with an intensity of 74 or 78 dB. Six combinations of prepulse and startle stimuli were employed (70-110, 74-110, 78-110, 70-120, 74-120, and 78-120). Six blocks of the nine trial types (prepulse/pulse: 0/0, 0/110, 0/120, 70/110, 74/110, 78/110, 70/120, 74/120, and 78/120) were presented in a pseudorandom order, such that each trial type was presented once within a block. The average inter-trial interval was 10-20 sec.

[Morris water maze test]

The conventional hidden platform version of the Morris water maze test was used to test spatial reference and working memory in mice and was performed using a modified procedure of a previous study [7]. The pool was 100 cm in diameter and the water was made opaque by using white water-based ink. The water temperature was maintained at room temperature (21-22 ˚C). The platform was 15 cm in diameter. Each mouse was trained with a black mark on the platform (visible training) and without the mark (hidden training) in four trials per day with inter-trial intervals for 30-40 min on average. In each trial, the mouse was allowed to swim until it found the platform, or until 60 sec had elapsed, at which point the mouse was guided to the platform. The mouse was then allowed to sit on the platform for 30 sec before it was picked up. The visible and hidden training were performed for four and six days, respectively. At 24 hr following the last hidden training session, the probe test was performed without the platform for 60 sec. Moreover, 7 days after the probe test, one more probe test was performed to evaluate the memory retention of the mice. All experiments were recorded, and the data were analyzed using Image WM software (O’Hara & Co.).

[Contextual and cued fear-conditioning test]

The ability of mice to learn and remember an association between environmental cues and aversive experiences was assessed by a contextual and cued fear-conditioning test. This test was performed with the minor modified procedure of a previous study [8]. The apparatus was “Contextual and cued fear conditioning test” (O’Hara & Co.). On day1, the conditioning session was performed, and on day 2, the context test and cued test sessions were performed. The conditioned stimulus (CS) and unconditioned stimulus (US) were 65 dB white noise and a 0.3 mA foot shock (2 sec), respectively. The detailed schedules and conditions were described in Fig. 10A.

**Results**

*[GAREM2 KO mice showed normal neuromuscular characteristics]*

In the neuromuscular test, whisker twitch, and ear twitch, reaching, key jangling, wire hang, and grip strength tests were performed. GAREM2 KO and WT mice exhibited the same level of grip strength (Fig. 1). GAREM2 KO mice were also normal in the other points including reaching, wire hang and so on.

*[GAREM2 KO mice exhibited normal levels of anxiety in the light/dark transition test]*

In the light/dark transition test, time spent in the light compartment is an index of low anxiety. The time spent in light compartment in GAREM2 KO mice did not differ to the WT mice (Fig. 2). Therefore, GAREM2 KO mice did not exhibit low anxiety. The latency to first transition (sec), transition number (times), and total distance (cm) in the dark or light chamber were also analyzed, and there was no difference between GAREM2 KO and WT mice (data not shown).

*[GAREM2 KO mice exhibited a normal level of nociception to pain]*

In the 55 ˚C hot plate test, GAREM2 KO and WT mice exhibited the same level of pain sensitivity. We deduced that this was because the onset of the hind paw response (sec) did not differ significantly between GAREM2 KO and WT mice (Fig. 3).

*[GAREM2 KO mice presented a normal level of working memory]*

Spontaneous alternation performance and working memory of GAREM2 KO mice were analyzed in the Y-maze test. The number of entries to the arms of apparatus and the alternation rate did not reveal any detectable difference between the two groups (Fig. 4).

*[GAREM2 KO mice demonstrated normal motor coordination and learning ability]*

GAREM2 KO mice were subjected to an evaluation of motor coordination and motor learning ability in the rotarod test. Latency to fall from the rotarod (sec) was analyzed, and GAREM2 KO mice tented to continue walking for longer before falling, compared with the WT mice (Fig. 5).

*[GAREM2 KO mice presented normal level of sociability and a preference for social novelty]*

In the three-chambered social interaction test, time spent near cage, number of entries to the chamber, and time spent in the chamber were analyzed, firstly in a box containing stranger and empty side chambers (Fig. 6A-C; sociability paradigm) and then a box containing stranger and familiar side chambers (Fig. 6D-F; social novelty preference paradigm). Both od WT and GAREM2 KO mice showed preference to “Stranger” compared to “Empty” and “Familiar” indicating that GAREM2 KO mice do not have deficit of sociability and preference for social novelty.

*[GAREM2 KO mice presented normal self-grooming activity]*

Self-grooming behavior have been considered as an index of one of the stress responses or repetitive behaviors [9]. The self-grooming time recorded manually with a stopwatch did not differ between GAREM2 KO and WT mice in the self-grooming test (Fig. 7).

*[GAREM2 KO mice showed normal sensory motor gating.]*

GAREM2 KO mice exhibited a normal startle response to the 110 dB and 120 dB startle stimuli (Fig. 8A). GAREM2 KO mice showed a normal PPI for the 70, 74, and 78 dB prepulse sound level followed by 110 and 120 dB in the startle response/prepulse inhibition test (Fig. 8B).

*[GAREM2 KO mice have normal levels of spatial learning memory]*

GAREM2 KO and WT littermates were also tested in the Morris water maze, which is frequently used to assess spatial reference memory in rodents. The learning ability was no different0 between GAREM2 KO and WT mice (Fig. 9A and B). Both genotypes mice spent significantly longer in the training quadrant compared with the other quadrants in the probe test performed after the training trials. GAREM2 KO mice showed no significant memory deficit in the probe trial (Fig. 9D-G). Moreover, the mice were tested with another probe test 7 days after the initial probe test to evaluate their memory retention. The results indicated that GAREM2 KO and WT mice had normal retention of memory (Fig. 9H-K).

*[GAREM2 KO mice presented normal contextual memory]*

The contextual and cued fear-conditioning test is the behavioral paradigm used to assess associative fear learning and memory in rodents. The test schedule and details of the fear-conditioning test is shown in Fig. 10A. Freezing criterion: 30 pixels, Freezing duration: 2sec. There was no difference in conditioning, context, altered context, and cued trials between GAREM2 KO and WT mice (Fig. 10B-D).

**
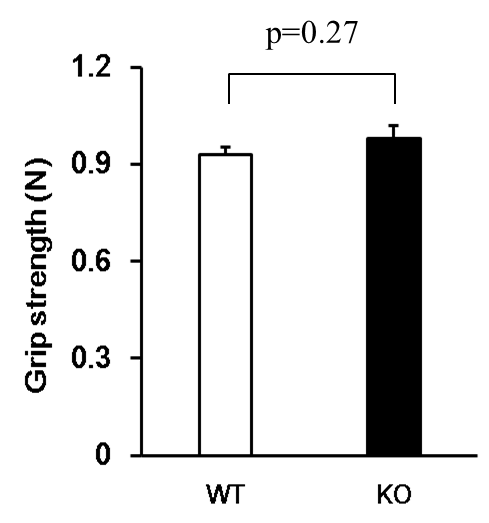
**

**Fig. 1 GAREM2 KO mice exhibited normal neuromuscular characteristics.** Graph showing the grip strength of forelimb in WT and GAREM2 KO mice. N=16 for each genotype and error bars indicate SEM.

**
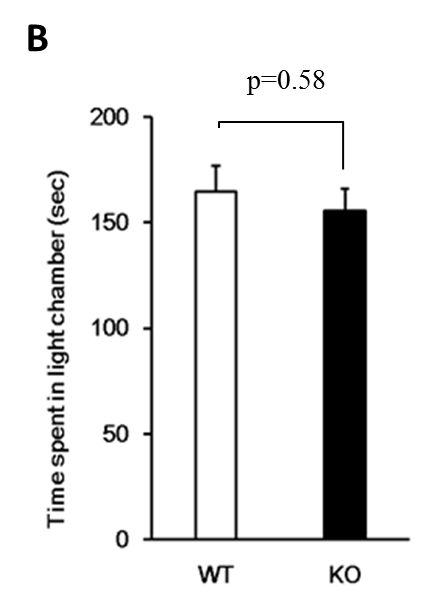
**

**Fig. 2 GAREM2 KO mice exhibit normal levels of**

**anxiety in the light/dark transition test**. Graph

showing the time spent in light chamber for each

genotype both GAREM2 KO and WT genotypes.

N=16 for each genotype and error bars indicate

SEM.


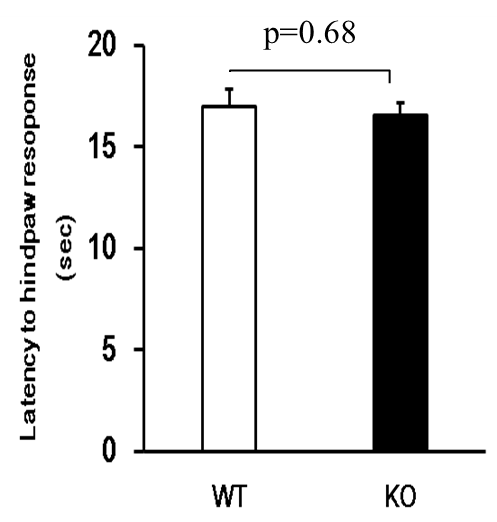


**Fig. 3 GAREM2 KO mice exhibited a normal level of nociception to pain in the 55 ˚C hot plate test.** Graph exhibiting the latency to hind paw response (sec) for both the GAREM2 KO and WT genotypes. N=16 for each genotype, error bars indicate SEM.


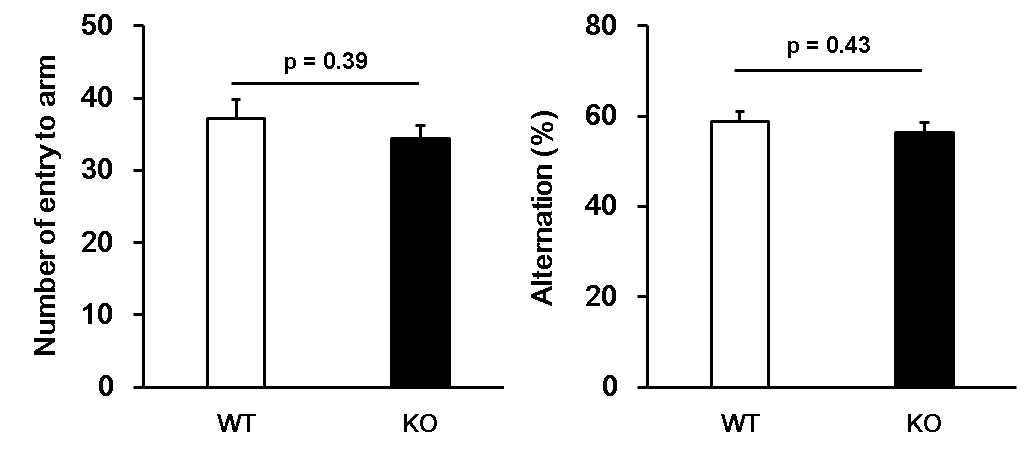


**A**

**A**

**B**

**A**

**Fig. 4 GAREM2 KO mice showed a normal level of working memory in the Y-maze test.** (A) Graph indicating the number of entries to the arms for each mouse strain. (B) Graph exhibiting the alternation rate (%) in WT and GAREM2 KO mice. N=16 for each genotype, error bars indicate SEM.


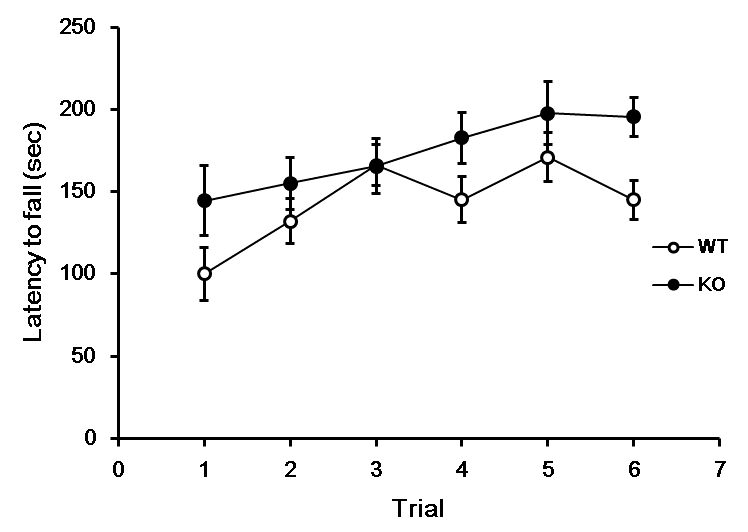

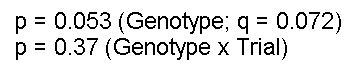
**Fig. 5 GAREM2 KO mice exhibited normal motor coordination and learning ability in the rotarod test.** The duration speed was from 4 to 40 rpm for 300 sec. Graph showing the analyzed latency to fall (sec) in WT and GAREM2 KO mice. N=16 for each genotype, error bars indicate SEM, two-way repeated measures ANOVA was used as statistical analysis.


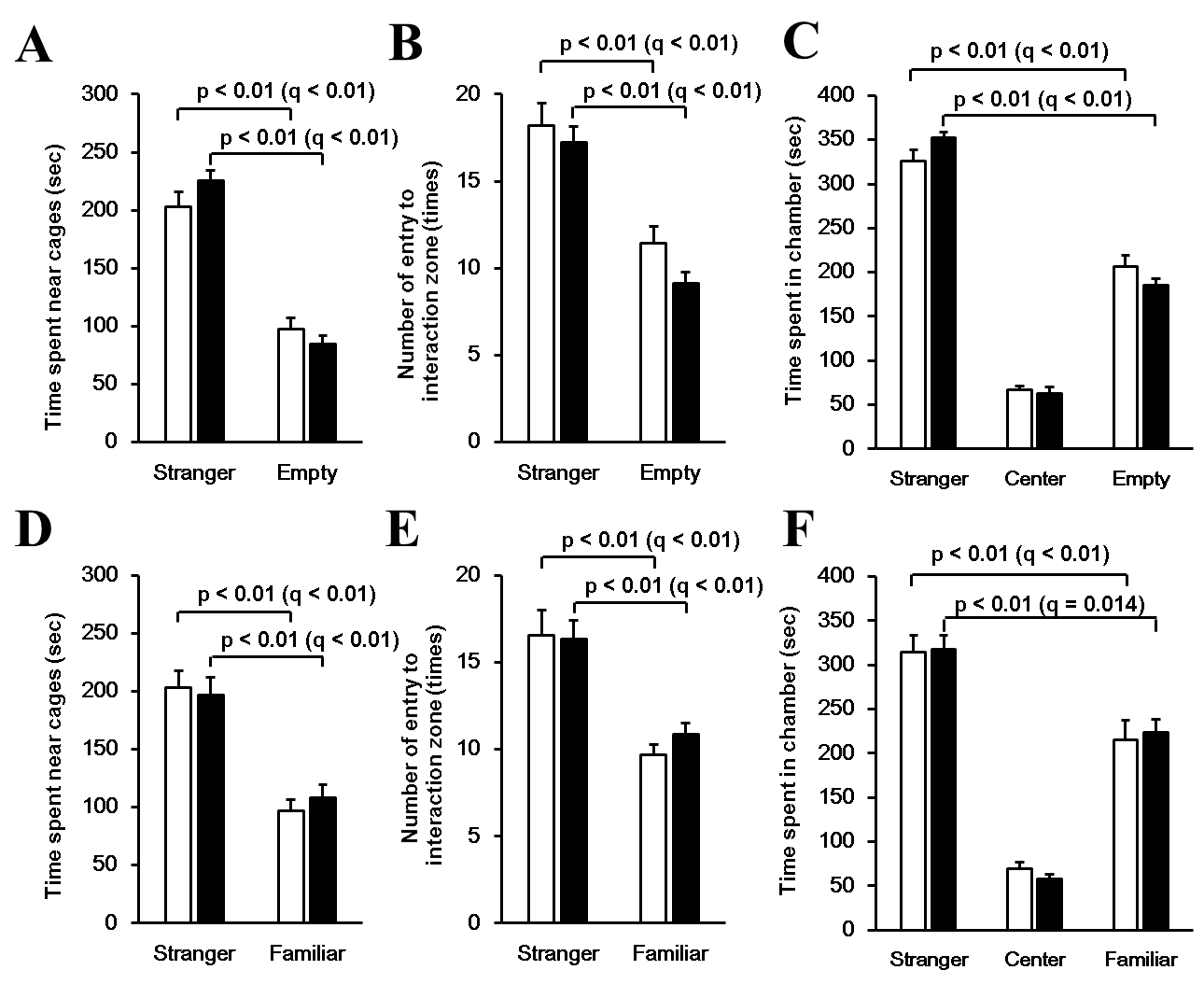
**Fig. 6. GAREM2 KO mice exhibited normal sociability and preference for social novelty in the three chamber social interaction test.** The white and black bars indicate WT and GAREM2 KO mice data, respectively. (A-C) Graphs showing the analyzed time spent near cage (sec), number of entries to chamber (times) and time spent in chamber (sec). (D-F) Graphs showing the same parameters as in A-C, but in a box containing stranger and familiar side chambers. A paired t-test was conducted to compare Stranger and Empty or Familiar chamber results. N=16 for each genotype, error bars indicate SEM. All p<0.01.


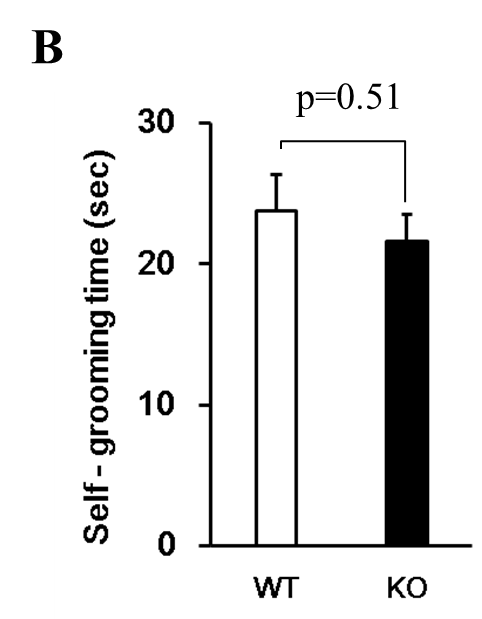
**Fig. 7 GAREM2 KO mice presented normal level of self-grooming behavior.** Graph indicating the analyzed self-grooming time (sec) for the GAREM2 KO and WT genotypes. N=16 for each genotype, error bars indicate SEM.

**A**

**A**

**B**

**A**

**
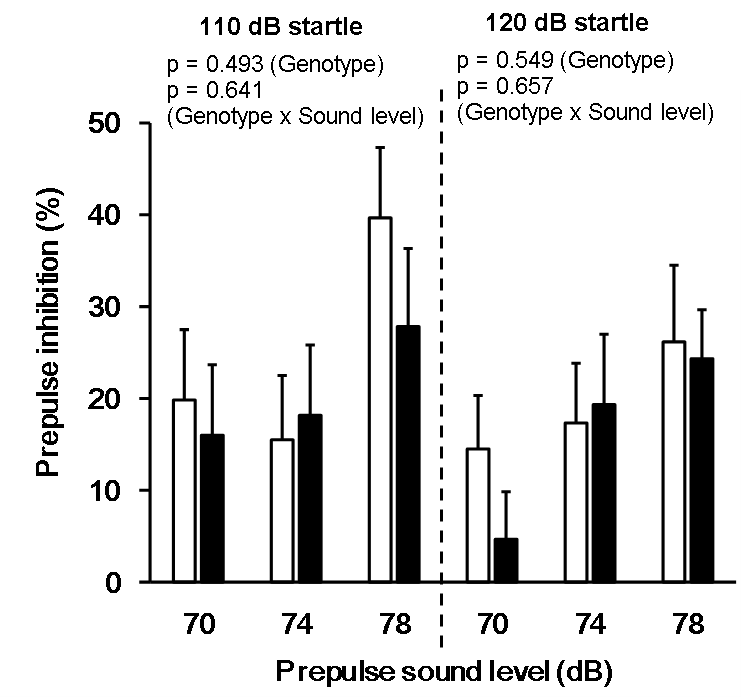
**

**
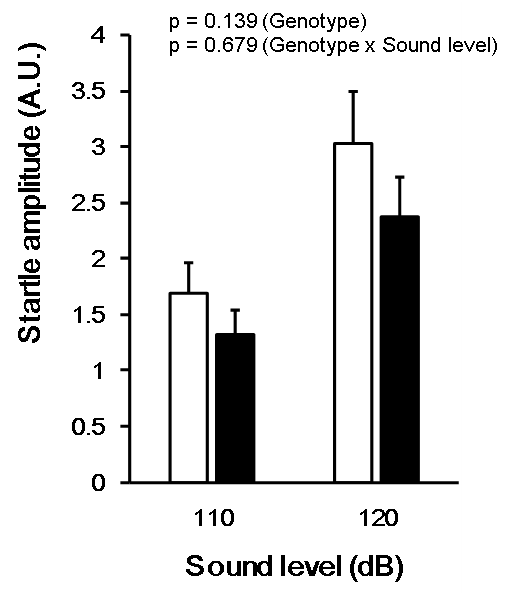
**

**Fig. 8 GAREM2 KO mice exhibited normal sensitivity for acoustic stimulus and sensory motor gating in prepulse inhibition test.** Test duration: 400sec, startle stimulus: 110 or 120dB, prepulse: 74 or 78 dB. The white and black bars indicate WT and GAREM2 KO data respectively. (A) The analyzed startle amplitude (arbitrary unit; A.U.) to evaluate acoustic response in GAREM 2 KO mice. (B) Graph exhibiting the prepulse inhibition (PPI, %) for each genotype. (PPI = 1- amplitudes of prepulse + startle / amplitudes of startle × 100). Error bars indicate SEM.

**C**

**A**

**A**

**A**

**B**

**A**

**C**

**A**

**D**

**A**

**E**

**A**


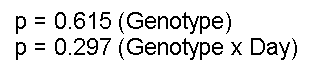

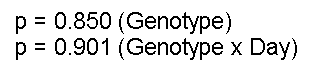


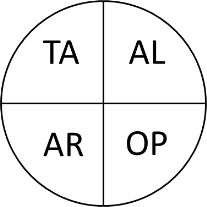

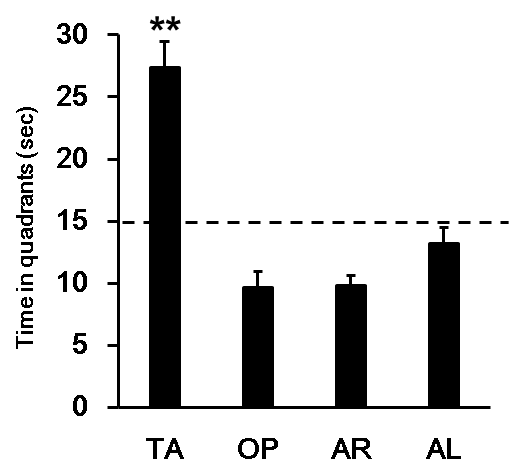

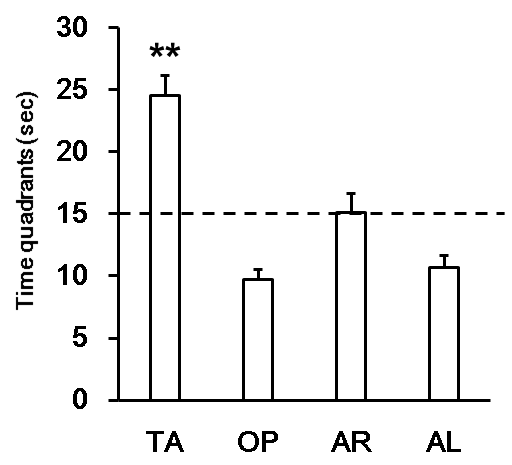

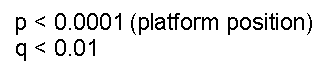

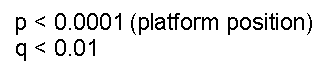


**GA**

**FGA**


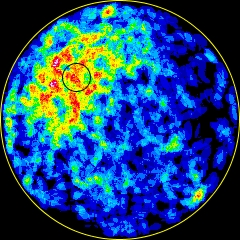

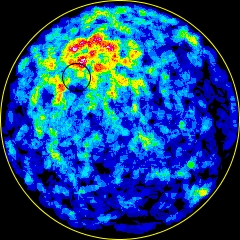


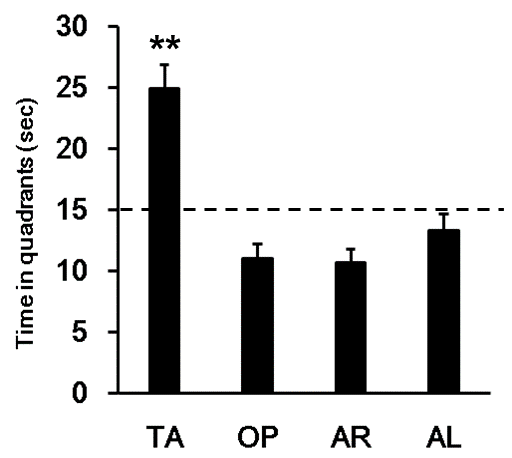

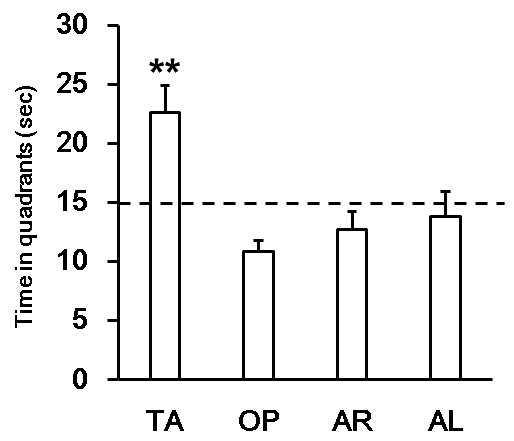


**H**

**A**

**I**

**A**


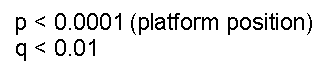

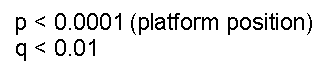


**J**

**A**

**K**

**A**


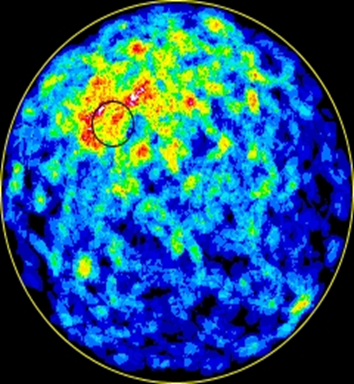

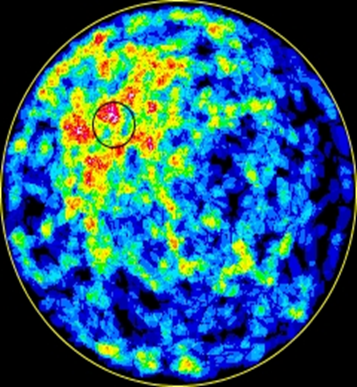


**Fig. 9 GAREM2 KO mice showed normal levels of the acquisition and retention of spatial learing memory in the MWM test.** The Morris Water Maze (MWM) test was performed in this schedule: visible for 4 days -hidden for 6 days -24hr elapsed –Probe test -7days elapsed -Probe test. (A) Graph showing the latency to arrive at the platform to assess the vision and/or motor of GAREM2 KO mice skills in the visible training period. (B) As before, in the hidden training period. (C) The areas of the test pool were divided to four areas, TA = the area containing the platform, OP = opposite side, AR = the right area of TA, AL = the left area of TA. (D and E) Graphs showing the time spent in quadrants in WT and GAREM2 KO mice, respectively, in the first probe test. p<0.0001, KO: F(3,60) = 33.6, p<0.0001. Newman-Keuls post hoc comparison; ** p<0.01. (F) Heat map exhibiting the frequency of staying in each area for WT mice. (G) As before, for GAREM2 KO mice. (H and I) As in D and E, for the second probe test (retention test, 7 days after the first probe test). WT: F(3,60) = 8.46, p<0.0001 , KO: F(3,60) = 22.10, p<0.0001. Newman-Keuls post hoc comparison; ** p<0.01. (J and K) As in F and G, heat maps for the retention test. N=16 for each genotype. Data from a KO mouse (m11) in the third trial of hidden period day 6 was omitted because of human error (improper platform position). Error bars indicate SEM. Dashed lines shown in (D, E, H, I) indicate 25% of the time (i.e. chance level).


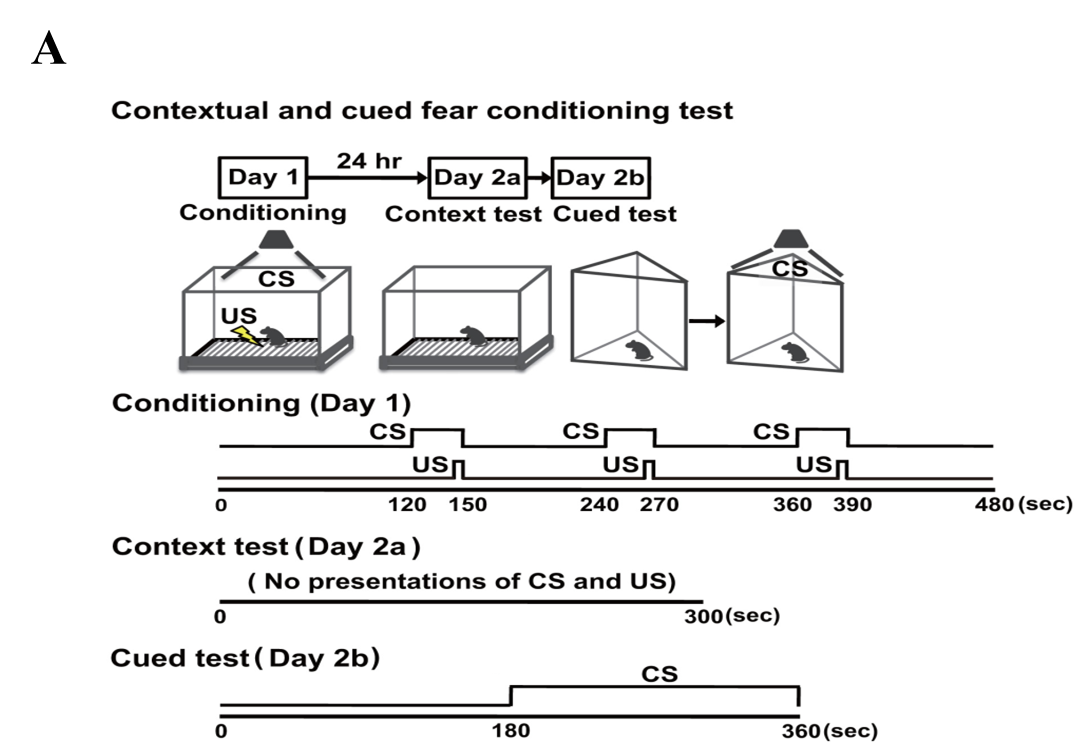


**
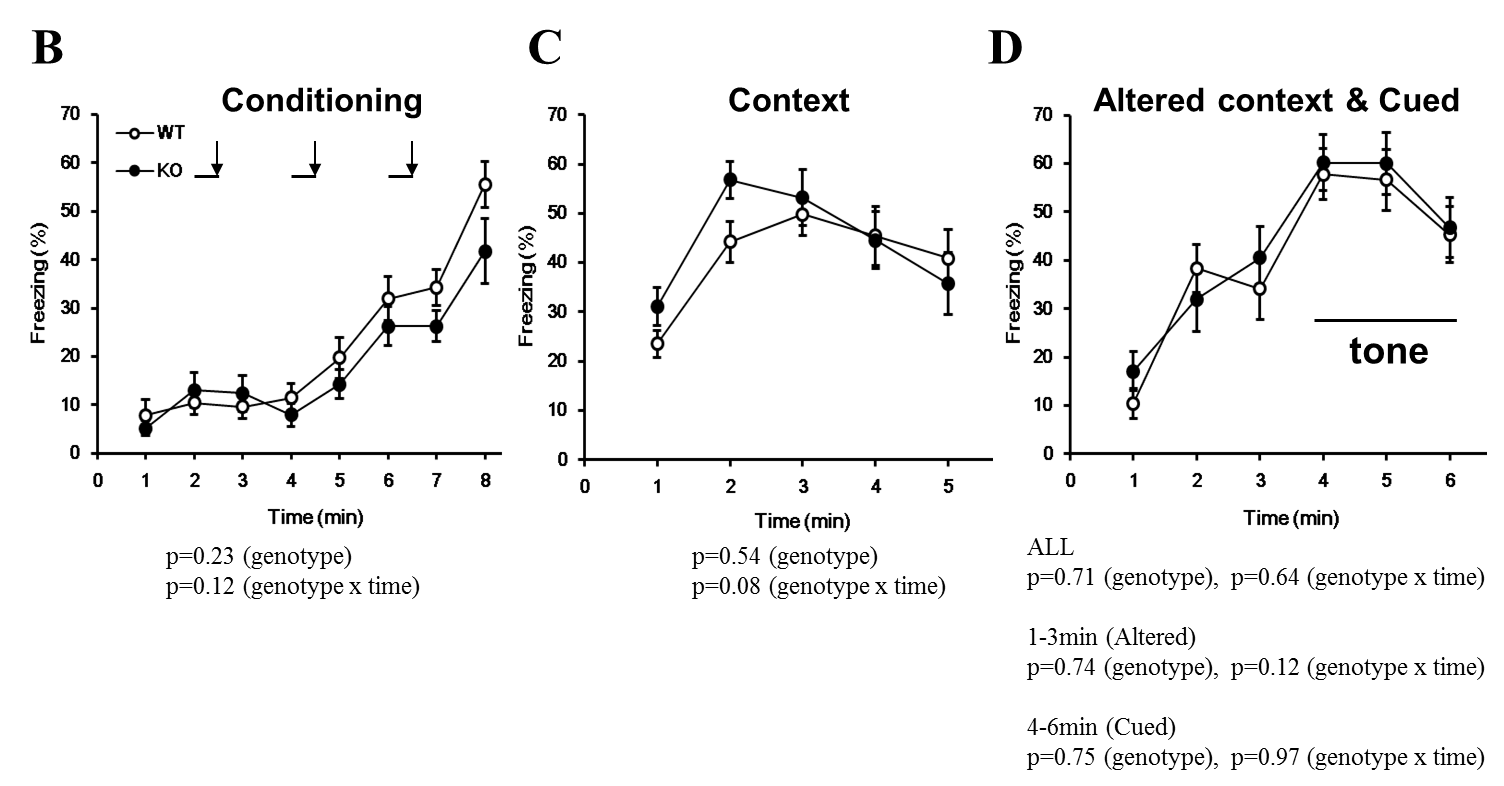
**

**Fig. 10 GAREM2 KO mice exhibited normal contextual memory in the contextual and cued fear-conditioning test. (A)** The schedule and details of the fear-conditioning test performed on GAREM2 KO and WT mice. Conditioned stimulus (CS): 65 dB white noise (30 sec), unconditioned stimulus (US): 0.3 mA electric foot shock (2 sec). (B) The freezing rate (%) of the mice in conditioning. (C) In context. (D) In altered context & cued phase. Black lines and arrows in upper graphs indicate tone (CS) and foot shock (US), respectively. N=16 for each genotype, error bars indicate SEM. A two way repeated measures ANOVA was performed.

**Reference**

[1] Tamada K, Tomonaga S, Hatanaka F, Nakai N, Takao K, Miyakawa T, et. al. Decreased Exploratory Activity in a Mouse Model of 15q Duplication Syndrome; Implications for Disturbance of Serotonin Signaling. PLoS ONE 2010;15:e15126.

[2] Miyakawa T, Yared E, Pak JH, Huang FL, Huang KP, Crawley JN. Neurogranin null mutant mice display performance deficits on spatial learning tasks with anxiety related components. Hippocampus 2001;11:763-75.

[3] Tsujimura A, Matsuki M, Takao K, Yamanishi K, Miyakawa T, Hashimoto-Gotoh T. Mice lacking the kf-1 gene exhibit increased anxiety- but not despair-like behavior. Front Behav. Neurosci. 2008;2:4.

[4] Takao K, Miyakawa T. Light/dark transition test for mice. J. Vis. Exp. 2006;104 p.

[5] Nadler JJ, Moy SS, Dold G, Trang D, Simmons N, Perez A, et al. Automated apparatus for quantitation of social approach behaviors in mice. Genes brain behave 2004;303-14.

[6] Leger M, Quiedeville A, Bouet V, Haelewyn B, Boulouard M, Schumann-Bard P et al. Object recognition test in mice. Nat Protocol. 2013;8:2531-7.

[7] Koshimizu H, Leiter LM, Miyakawa T. M4 muscarinic receptor knockout mice display abnormal social behavior and decreased prepulse inhibition. Mol Brain. 2012;5:10.

[8] Shoji H, Takao K, Hattori S, Miyakawa T. Contextual and Cued Fear Conditioning Test Using a Video Analyzing System in Mice. J. Vis. Exp. 2014;85.

[9] Kalueff AV, Stewart AM, Song C, Berridge KC, Graybiel AM, Fentress JC Neurobiology of rodent self-grooming and its value for translational neuroscience. Nat Rev Neurosci. 2016;17:45-59.
